# Patients with laboratory evidence of West Nile virus disease without reported fever

**DOI:** 10.1017/S0950268819001079

**Published:** 2019-06-17

**Authors:** K. Landry, I. B. Rabe, S. L. Messenger, J. K. Hacker, M. L. Salas, C. Scott-Waldron, D. Haydel, E. Rider, S. Simonson, C. M. Brown, S. C. Smole, D. F. Neitzel, E. K. Schiffman, A. K. Strain, S. Vetter, M. Fischer, N. P. Lindsey

**Affiliations:** 1 Arboviral Diseases Branch, Centers for Disease Control and Prevention, Fort Collins, CO, USA; 2 Viral and Rickettsial Disease Laboratory, California Department of Public Health, Richmond, CA, USA; 3 Louisiana Office of Public Health, Baton Rouge, LA, USA; 4 Massachusetts Department of Public Health, Jamaica Plain, MA, USA; 5 Minnesota Department of Health, Saint Paul, MN, USA

**Keywords:** Arboviruses, epidemiology, West Nile virus

## Abstract

In 2013, the national surveillance case definition for West Nile virus (WNV) disease was revised to remove fever as a criterion for neuroinvasive disease and require at most subjective fever for non-neuroinvasive disease. The aims of this project were to determine how often afebrile WNV disease occurs and assess differences among patients with and without fever. We included cases with laboratory evidence of WNV disease reported from four states in 2014. We compared demographics, clinical symptoms and laboratory evidence for patients with and without fever and stratified the analysis by neuroinvasive and non-neuroinvasive presentations. Among 956 included patients, 39 (4%) had no fever; this proportion was similar among patients with and without neuroinvasive disease symptoms. For neuroinvasive and non-neuroinvasive patients, there were no differences in age, sex, or laboratory evidence between febrile and afebrile patients, but hospitalisations were more common among patients with fever (*P* < 0.01). The only significant difference in symptoms was for ataxia, which was more common in neuroinvasive patients without fever (*P* = 0.04). Only 5% of non-neuroinvasive patients did not meet the WNV case definition due to lack of fever. The evidence presented here supports the changes made to the national case definition in 2013.

## Introduction

West Nile virus (WNV), a mosquito-borne flavivirus, is a leading cause of arboviral disease in the USA. The virus was first identified in North America in 1999 and has since become endemic to the USA, where it causes annual seasonal outbreaks [1, 2]. Most WNV infections are asymptomatic; of those who become ill, most have a self-limited febrile illness. Other common symptoms include headache, myalgia, arthralgia, vomiting, diarrhoea, or maculopapular rash [3, 4]. Less than 1% of infected persons develop neuroinvasive disease, which can include encephalitis, meningitis, acute flaccid paralysis [5–7]. The Council of State and Territorial Epidemiologists (CSTE) collaborates with the Centers for Disease Control and Prevention (CDC) to define nationally notifiable diseases, including arboviruses like WNV. Starting in 2004, the CSTE case definition for arboviral diseases included documented fever as a required clinical criterion for both neuroinvasive and non-neuroinvasive arboviral disease [8]. After this case definition was implemented, some state and local epidemiologists expressed concerns about patients exhibiting clinical symptoms with convincing laboratory evidence for a diagnosis of WNV infection but without fever and therefore not meeting the CSTE case definition.

In 2013, CSTE revised the case definition by removing fever as a clinical requirement for arboviral neuroinvasive disease and allowing any measured or subjective fever to meet the clinical criteria for non-neuroinvasive disease [9]. Although the revised case definition was implemented in 2014, little is known about how often patients with a symptomatic illness and laboratory evidence of WNV infection lack a reported fever. The aims of this project were to determine how often this occurs and evaluate differences in the clinical findings or laboratory evidence of WNV infection between patients with and without fever.

## Methods

Enhanced case investigations were conducted in four states (California, Louisiana, Massachusetts and Minnesota) for patients who met the confirmed or probable laboratory criteria in the case definition in 2014. Asymptomatic patients were excluded. The enhanced case investigations included the collection of clinical and laboratory data from healthcare providers and patients.

State and local health departments routinely report WNV disease cases that meet the national CSTE case definition to CDC's arboviral surveillance system, ArboNET. For this project, case-patients meeting the case definition for either neuroinvasive or non-neuroinvasive disease was reported to ArboNET, as well as case-patients who did not meet the CSTE case definition but were eligible for this project due to their laboratory test results. ArboNET variables typically include age, sex, race, country and state of residence, date of illness onset, case status, clinical syndrome, whether the patient was hospitalised and if death occurred due to illness. All data collected through the enhanced case investigations were also reported via ArboNET.

In order to obtain a conservative estimate of the numbers of patients lacking fever, all patients with measured or subjective fever or chills were considered to have fever in this analysis. Demographics, clinical symptoms and laboratory evidence were compared between patients with and without fever. Comparisons were made for patients with neuroinvasive and non-neuroinvasive clinical presentations separately. Categorical variables were summarised using counts and proportions and compared using Fisher's exact test; continuous variables were summarised using median and range and compared using the Wilcoxon rank-sum test. The data were analysed using SAS version 9.4 (Cary, NC).

## Results

A total of 977 patients who met the confirmed or probable laboratory criteria for WNV disease were reported from the four states during the project period. Of those, 956 (98%) had data reported on the presence or absence of fever and were included in the analysis. Among the 956 patients, 823 (86%) had a measured fever, 94 (10%) had subjective fever or chills and 39 (4%) had no reported fever or chills. Of the 39 patients without measured or subjective fever, 16 (41%) met the confirmed laboratory criteria in the case definition and 23 (59%) met the probable criteria. Twenty-three (59%) of the 39 patients without reported fever had neurologic symptoms and were classified as neuroinvasive disease cases. The remaining 16 patients did not meet the clinical criteria of the case definition due to lack of fever and symptoms consistent with neuroinvasive disease and were therefore not counted as cases in national surveillance data. Overall, seven (18%) of the 39 patients without fever reported taking antipyretics (e.g. acetaminophen, aspirin, or other non-steroidal anti-inflammatory medications); one of the seven patients also was immunosuppressed. Four of the seven patients without fever reported symptoms consistent with neuroinvasive disease.

Overall, 620 (65%) of the 956 patients included in the analysis were reported to have neuroinvasive disease. Of those 620, 597 (96%) were reported to have fever. Among the 23 patients without fever, 14 (61%) met the confirmed laboratory criteria, including 12 patients with WNV IgM antibodies in cerebrospinal fluid, one patient with WNV IgM and neutralizing antibodies in serum and one patient with WNV RNA in serum. There were no significant differences in age, sex, or proportion meeting the confirmed laboratory criteria between patients with and without fever (Table 1). However, 578 (97%) of 597 patients with febrile neuroinvasive disease were hospitalised compared to 18 (78%) of the 23 patients without reported fever (*P* < 0.01). Ataxia was reported in 3% (18/597) of patients with fever and neurologic symptoms compared to 13% (3/23) of patients with neuroinvasive disease symptoms but no reported fever (*P* = 0.04).

**Table 1.** Characteristics of case-patients with laboratory evidence of West Nile virus infection, by the presence of fever and neuroinvasive signs/symptoms

	Neuroinvasive^a^					Non-neuroinvasive				
	No fever		Any fever		*P*-value^b^	No fever		Any fever		*P*-value^b^
	(*n* = 23)		(*n* = 597)			(*n* = 16)		(*n* = 320)		
	No.	(**%)**	No.	(**%)**		No.	(**%)**	No.	(**%)**	
Male sex	15	(65)	403	(68)	0.82	6	(38)	189	(59)	0.12
Median age (range)	57	(19–84)	57	(3–94)	0.29^c^	55	(15–87)	55	(5–89)	0.86^c^
Hospitalised	18	(78)	578	(97)	<0.01	2	(13)	159	(50)	<0.01
Laboratory confirmed^d^	14	(61)	360	(60)	1.00	2	(13)	54	(17)	1.00
Clinical symptoms										
Rash	6	(26)	112	(19)	0.41	7	(44)	100	(31)	0.29
Headache	14	(61)	409	(69)	0.49	12	(75)	205	(64)	0.43
Diarrhoea	4	(17)	131	(22)	0.80	3	(19)	68	(21)	1.00
Nausea/vomiting	12	(52)	345	(58)	0.67	5	(31)	163	(51)	0.20
Myalgia	11	(48)	245	(41)	0.53	11	(69)	194	(61)	0.61
Arthralgia	2	(9)	109	(18)	0.40	6	(38)	94	(29)	0.58
Arthritis	2	(9)	104	(17)	0.40	3	(19)	84	(26)	0.77
Stiff neck	4	(17)	210	(35)	0.12	4	(25)	76	(24)	1.00
Paresis/paralysis	5	(22)	112	(19)	0.79	--		--		
Altered mental status	13	(57)	316	(53)	0.83	--		--		
Seizures	1	(4)	33	(6)	1.00	--		--		
Ataxia	3	(13)	18	(3)	0.04	--		--		
Parkinsonism/cogwheel rigidity	1	(4)	1	(<1)	0.07	--		--		

^a^Includes altered mental status, seizures, ataxia, or parkinsonism/cogwheel rigidity.

^b^Fisher's exact.

^c^Wilcoxon rank-sum test.

^d^Demonstration of specific viral antigen or nucleic acid in tissue, blood, or CSF, ⩾fourfold increase in virus-specific quantitative antibody titers in paired sera, virus-specific IgM antibodies in serum with confirmatory virus-specific neutralizing antibodies in the same or a later specimen, or virus-specific IgM antibodies in CSF.

Among the 336 patients without neuroinvasive disease symptoms, 320 (95%) were reported to have a fever. Among the 16 patients without fever, only two (13%) met the confirmed laboratory criteria with WNV RNA detected in blood, including one patient identified through routine blood donor screening who subsequently developed clinical symptoms. There were no significant differences in age, sex, or proportion meeting the confirmed laboratory criteria between non-neuroinvasive disease patients with and without fever (Table). Of significance, 159 (50%) of the 320 patients with febrile non-neuroinvasive disease were hospitalised compared to two (13%) of the 16 patients without reported fever (*P* < 0.01).

## Discussion

These data indicate that WNV disease without fever is uncommonly reported but does occur. Overall, only 4% of patients with laboratory-confirmed or probable WNV infection did not have a measured or subjective fever. Most afebrile patients did not have a recorded medical condition or medication use that could potentially explain the absence of fever.

Surveillance case definitions are used to classify and count cases consistently across reporting jurisdictions and are not intended to be used by healthcare providers for making clinical diagnoses or determining treatment plans for particular patients. While high sensitivity and specificity of case definitions are desirable, generally one comes at the expense of the other. In this project, the majority of afebrile patients with neuroinvasive disease symptoms met the confirmed laboratory criteria in the case definition, increasing the confidence in the diagnosis of an acute WNV infection. In addition, other clinical features reported for neuroinvasive disease patients were similar among febrile and afebrile cases. These findings suggest that the case definition is precise enough to correct classify these cases without the requirement of the fever criterion in the case definition for neuroinvasive disease.

Among patients without neuroinvasive disease symptoms, only 5% did not meet the clinical criteria because of lack of fever and were not counted as cases in national surveillance data. If this percentage were applied to the national WNV data, an estimated 43 patients would have met the confirmed or probable laboratory criteria for WNV disease in 2014 but would not have been counted as cases in national surveillance data. The laboratory evidence for most of these patients was not as convincing as for the neurologic cases; 14 of the 16 had only a single IgM positive antibody test in serum.

Clinical presentation and laboratory evidence of infection among patients with and without fever were similar for those with and for those without neuroinvasive disease symptoms. The only identified difference in the clinical presentation was for ataxia, which was significantly more common among neuroinvasive disease patients without fever than among those with fever. The potential reasons for this difference are unknown. Among both neuroinvasive and non-neuroinvasive patients, those with fever were significantly more likely to be hospitalised than those without fever. This could be related to either true or perceived differences in the severity of the disease, or the need to evaluate and treat other possible etiologies.

Previous studies have suggested that symptomatic WNV infection can occur without fever. A study conducted by the American Red Cross assessed symptoms attributed to WNV infection by comparing symptom frequency among blood donors with confirmed early WNV infection and those with an initial reactive test that was unconfirmed [10]. One notable finding of that study was the absence of reported fever among a substantial proportion (44%) of the symptomatic persons with confirmed WNV infection. Additionally, a number of reports summarizing WNV disease cases identified through passive surveillance have reported patients with symptomatic WNV infection without fever, with percentages of afebrile patients ranging from 19% to 37% [11–13]. The percentages of afebrile patients in these studies might differ from that calculated in our analysis due to a number of reasons, including the study population, definition of fever, method of data collection and laboratory evidence that the patient's current symptoms were attributable to WNV infection. Additionally, these previous studies were performed in the context of WNV outbreaks rather than average seasons (as included in our study). During outbreak years, it is likely that a greater number of people are tested, possibly leading to enhanced identification of laboratory evidence of WNV infection in less severely ill people, including those that might not have fever.

There are a number of limitations to this analysis. Patients with subjective fever and chills were combined with those with measured fever, possibly resulting in an underestimate of the percentage of afebrile WNV disease patients. Almost 90% of febrile cases had documented, measured fevers, so this is unlikely to have substantially impacted our results. Although asymptomatic infections were excluded from the analysis, it is possible that some of the symptoms reported by patients without fever were unrelated to WNV infection. Additionally, patients with only a positive WNV IgM result in serum might not have had an acute WNV infection, since this may reflect false-positivity or antibody persistence following previous infection [14]. The WNV infections included in this analysis were identified through passive disease surveillance, which is known to underestimate the true prevalence of disease. To be reported to public health, the patient must seek care, a clinician must request appropriate diagnostic tests and healthcare providers or laboratories must then report cases to public health authorities. It does not seem likely that the presence or absence of fever would impact whether or not a positive laboratory test is reported, but could impact whether or not a clinician orders a WNV test. Finally, the data presented here are from four states and may not be representative of all cases nationally.

In summary, in this study population, WNV disease without fever was relatively uncommon. Among neuroinvasive disease cases, the clinical and laboratory evidence among cases without fever support the removal of that requirement from the case definition. Overall, only a small proportion of non-neurologic patients failed to meet the case definition because of lack of fever and the laboratory evidence for those cases was less convincing. If the percent of cases missed were applied to national surveillance data, the difference was not substantial, suggesting that a change in case definition to remove the requirement of fever for non-neuroinvasive disease is unnecessary.
